# Commentary: Commentary: Beauty Requires Thought

**DOI:** 10.3389/fpsyg.2018.01917

**Published:** 2018-10-09

**Authors:** Katharina Bluehm

**Affiliations:** Independent Researcher, Berlin, Germany

**Keywords:** beauty, physical attractiveness, immanent teleology, Kant, self-organization, aesthetic judgment, neuroaesthetics

I wish to make sense of an obvious conflict between a recent study published in Current Biology (Brielmann and Pelli, 2017) and a commentary on this study published in *Frontiers in Psychology* (Luoto, 2017). Accordingly, I comment on both the commentary and the study equally.

The Kant-inspired claim that “Beauty Requires Thought,” experimentally confirmed by the study of Brielmann and Pelli (2017) and Luoto (2017) succinctly argued objection, insisting that elaborate “cognitive processing is not a prerequisite for the appreciation of beauty” are diametrically opposed. There is, however, a possibility to do justice to both lines of evidence and to turn the contradiction into a contribution.

Kant's third Critique has two parts, the *Critique of the Aesthetic Power of Judgment* and the *Critique of the Teleological Power of Judgment*. The latter is about the “objective purposiveness of nature in its entirety, as a system of which the human being is a member” (Kant, 1790, 05:380). Objective purposiveness makes an appreciation “either teleological or grounded in mere sensations of an object (gratification or pain)” (Kant, 1790, 05:270, cf. 279). Responding in the horizon of reproduction and care is inherently purposive and predisposed by instincts and inclinations. Instinct is “[t]he inner necessitation of the faculty of desire to take possession of [an] object before one is familiar with it” (Kant, 1798, 07:365). Inclinations are acquired by experience. “In some cases (e.g., the sexual instinct; see Kant, 1775-1776, 25:584) one can feel an instinct without a direct cognition of the object of that instinct.” (Frierson, 2014) This strikingly coincides with the evidence Luoto provides concerning sexual stimuli (Ponseti and Bosinski, 2010; Gillath and Collins, 2016), facial beauty (Willis and Todorov, 2006) and the integration of visual information outside of consciousness (Mudrik et al., 2014; cf. Kant, 1798, 07:135f).

*Qualitative* (that is, *formal*) perfection is “belonging to *teleology”* (Kant, 1797, 06:386). Accordingly, Luoto's line of evidence has also an overlap with the judgment of *perfection*, an aesthetic judgment adherent to a prototype. This is especially interesting since Armstrong and Detweiler-Bedell (2008) connected this type of judgment to the fluency model of aesthetic pleasure (Reber et al., 2004).

Nonetheless, beyond responding to physical attractiveness in an implicitly teleological manner I can as well adopt a solely aesthetic-reflective attitude—also in the face of physical attractiveness. Here “at issue is not … what it is for us as a purpose, but how we take it in” (Kant, 1790, 05:350). The course of the aesthetic-reflective engagement reflects the richness of the expressive patterns and possible interpretations, and manifests itself in an animated interplay of imagination and understanding. The inherent motivational dynamics of the interplay are pleasant (Bluehm, 2017). Different stimuli in this way induce the same type of pleasure. Crucially, cognitive faculties are involved—nonetheless beauty is felt, not concluded (Wieland, 2001; Bluehm, 2018). Pleasure (inter alia) is explained “as the feeling that expresses a condition that promotes life and its activity.” (Guyer, editorial note, p. 366 in Kant, 1790; cf. 05:204, 05:244, 05:313). So the pure (or reflective) aesthetic judgment is triggered by the purposiveness of the form of an object (Kant, 1794, 20:230) for cognition in general (roughly, being “richly interpretable”—Biederman and Vessel, 2006; Biederman, 2017) without to serve any specific purpose. This type of purposiveness is said to pertain to the community of all judging subjects, understood in the most general sense (united e.g., by space and time as common format of intuition).

In conclusion, both lines of evidence, Brielmann/Pelli's *and* Luoto's, seem to have their right. They refer to different phenomena. The obvious conflict between Brielmann/Pelli and Luoto supports the assumption, that the correspondence between aesthetic preference behavior (as life world phenomenon) and aesthetic-reflective practice is rather limited.

Addison (1712) already distinguishes “two kinds of beauty”, the first with background in sexual desire (Guyer, 2015). In the Kantian approach (1790) different phenomena obtain different names. Levinson (2011) posits “Beauty Is Not One.” Empirically, Brielmann et al. (2017) found, artistic coherence is not experienced to depend on idealized body proportions. Schulz and Hayn-Leichsenring (2017) dissociate attractiveness and artistic beauty.

Besides the valuable overall outcome, Brielmann and Pelli's presentation of their Kantian sources prompts a word of warning. It is the *imagination*, which enters into a harmonious interplay with the understanding. To ascribe the free play to “the faculties of sensation and understanding ([2] (p. 102, §4), [7])” is mistaken (Brielmann and Pelli, 2017 p. 1506). Sensations are what the faculty of sensibility—a receptive opposed to a spontaneous faculty—receives from the object. Further: 1764, in the *Observations on the feeling of the beautiful and the sublime* Kant by no means “claimed that beauty requires thought and that sensuous pleasures do not” (Brielmann and Pelli, 2017, p. 1506). To the contrary, in this early essay beauty is addressed as “agreeable,” and is said to “charm” and to “touch” (Kant, 1764, 02:208f). All these attributes become *contrast terms* to beauty in Kant's mature approach, and furnish exactly the realm, which Brielmann and Pelli endeavor to test as their second task: whether sensuous pleasures can be experienced as beautiful. Thus, they included trials with primarily sensory stimuli, such as candies. Sensuous pleasures are for Kant “the agreeable” and do not “supply a supplement … to the satisfaction in the form.” However, they can contribute indirectly by “awakening and sustaining attention” (Kant, 1790, 05:225f). Beauty permeated with sensory satisfaction is called *impure*. In the experiment, 37% of participants “responded ‘definitely yes' for beauty on candy trials.” The authors report, that most of these participants “remarked that sucking candy had personal meaning for them, like a fond childhood memory” (Brielmann and Pelli, 2017, p. 1511). The taste seem to operate as memory key (Proust, 1913-1927). We see an attention-directing role in addition to the sensory pleasure itself. One of the notable merits of the paper is the fact that it records the natural use of the term “beautiful” for quite protean animating immersive positive experiences.

## Author contributions

The author confirms being the sole contributor of this work and has approved it for publication.

### Conflict of interest statement

The author declares that the research was conducted in the absence of any commercial or financial relationships that could be construed as a potential conflict of interest.
